# Prognostic Nomogram and Predictive Factors in Refractory or Relapsed Diffuse Large B-Cell Lymphoma Patients Failing Front-Line R-CHOP Regimens

**DOI:** 10.7150/jca.36997

**Published:** 2020-01-14

**Authors:** Shiyu Jiang, Yan Qin, Peng Liu, Jianliang Yang, Sheng Yang, Xiaohui He, Shengyu Zhou, Lin Gui, Changgong Zhang, Liqiang Zhou, Yan Sun, Yuankai Shi

**Affiliations:** Department of Medical Oncology, National Cancer Center/National Clinical Research Center for Cancer/Cancer Hospital, Chinese Academy of Medical Sciences & Peking Union Medical College, Beijing Key Laboratory of Clinical Study on Anticancer Molecular Targeted Drugs, Beijing, 100021, China.

**Keywords:** diffuse large B-cell lymphoma, overall survival, nomogram, efficacy

## Abstract

**Background**: The clinical course of relapsed or refractory (r/r) diffuse large B-cell lymphoma (DLBCL) is variable, with limited efficacy data of second-line treatment in a post-rituximab real-world context. Hence, we explored the predictors and constructed a nomogram for risk stratification in this population.

**Patients and methods**: Among 296 r/r DLBCL patients pretreated with R-CHOP (rituximab plus cyclophosphamide, doxorubicin, vincristine, and prednisone) at the Cancer Hospital, Chinese Academy of Medical Sciences & Peking Union Medical College between 2006 and 2017, 231 were included for nomogram construction. After randomization, we constructed the prognostic nomogram in the primary cohort (n=161) based on a multivariate analysis and confirmed it in the validation cohort (n=70). Additionally, we explored predictive factors for second-line therapy using a ordinal regression analysis.

**Results**: Four independent prognostic factors including rituximab in the second-line setting, initial Eastern Cooperative Oncology Group (ECOG) performance status (PS), response to front-line treatment, and invasion on progression/recurrence were used to construct the nomogram. The nomogram had a C index of 0.70 with AUC values of 0.73 and 0.71 for the primary and validation cohorts, respectively. Subsequently, three risk groups (low, intermediate, and high) were determined with median overall survival (OS) of 38.0, 25.0, and 7.0 months, respectively. Additionally, we conducted a multivariate ordinal regression analysis and identified prior response to front-line treatment (odds ratio=4.50, 95% CI: 1.84-11.27, p=0.001) and bulky disease at diagnosis (odds ratio=0.36, 95% CI: 0.182-0.702, p=0.003) were two independent factors for second-line treatment efficacy.

**Conclusions**: The established predictors for treatment efficacy and the novel nomogram for survival in r/r DLBCL patients could potentially be applied for risk stratification and treatment guidance in the post-rituximab era.

## Introduction

Diffuse large B-cell lymphoma (DLBCL) represents the most common subset of non-Hodgkin lymphoma (NHL), accounting for approximately 40% of NHL burden in China 1. The R-CHOP (rituximab plus cyclophosphamide, doxorubicin, vincristine, and prednisone) regimen, as the standard first-line treatment, can cure over one-half of patients. Nevertheless, 30-40% of patients will experience refraction or relapse and have poor prognosis, especially for those refractory to first-line immunochemotherapy with an overall response rate (ORR) of 26% and a complete response (CR) rate of 7% 2-4. Second-line chemotherapy and high-dose therapy (HDT) followed by autologous stem cell transplantation (ASCT) offer an opportunity of cure for this population 5-6. However, comorbidities, chemosensitivity, and financial support precludes the utility and availability of ASCT. Thus, survival for the refractory or relapsed (r/r) population remains poor worldwide.

Although rituximab yields significant benefits for patients with DLBCL and has been incorporated in front-line treatment for nearly 2 decades 7, the impact of rituximab on patient outcomes during second-line treatment is less consistent compared with that in the first-line setting 8-9. Moreover, prior evidence showed that exposure to rituximab during front-line treatment was associated with a worse outcome with the second-line treatment 8,10.

The International Prognostic Index (IPI) constructed from the disease stage, age, serum concentrations of lactate dehydrogenase (LDH), Eastern Cooperative Oncology Group (ECOG) performance status (PS), and number of extranodal sites has been the standard tool for risk stratification and treatment guidance since the 1990s 11. With the introduction of rituximab, revisions of the IPI have been applied, such as the Revised IPI (R-IPI) 12 and National Comprehensive Cancer Network IPI (NCCN-IPI) 13. In addition to the achievements in the prognostication of de novo DLBCL, prognostic parameters for r/r patients have also been explored in the era of rituximab. The Secondary Age-Adjusted IPI (saaIPI) was determined by the absence or presence of risk factors including poor performance status, elevated LDH, and advanced stage before second-line treatment 14. From the CORAL study data, early relapse (time from diagnosis to relapse of less than 12 months), prior rituximab exposure, and the saaIPI were negatively correlated with the response to second-line treatment as well as overall survival (OS) 5.

Unlike traditional models such as the IPI and saaIPI, the nomogram is a statistical predictive model in a visual format for determining the points of each variable value that provides improved predictive accuracy for clinical outcomes. Previously, the nomogram was demonstrated in several malignancies 15-18 as well as de novo DLBCL 19, which shows the accurate estimate of patient survival. Given the paucity of prognostication in patients with r/r DLBCL, there is a need for more accurate prognostic tools in efficacy with the currently available treatment options and patient outcomes. Herein, we conducted a retrospective study of r/r DLBCL cases pretreated with the R-CHOP regimen to (i) evaluate the clinical features and treatment efficacy, (ii) identify the prognostic indicators for survival and efficacy of second-line therapy, and (iii) develop the first nomogram for survival prediction in r/r DLBCL.

## Patients and methods

### Patients and study design

**Fig 1:**
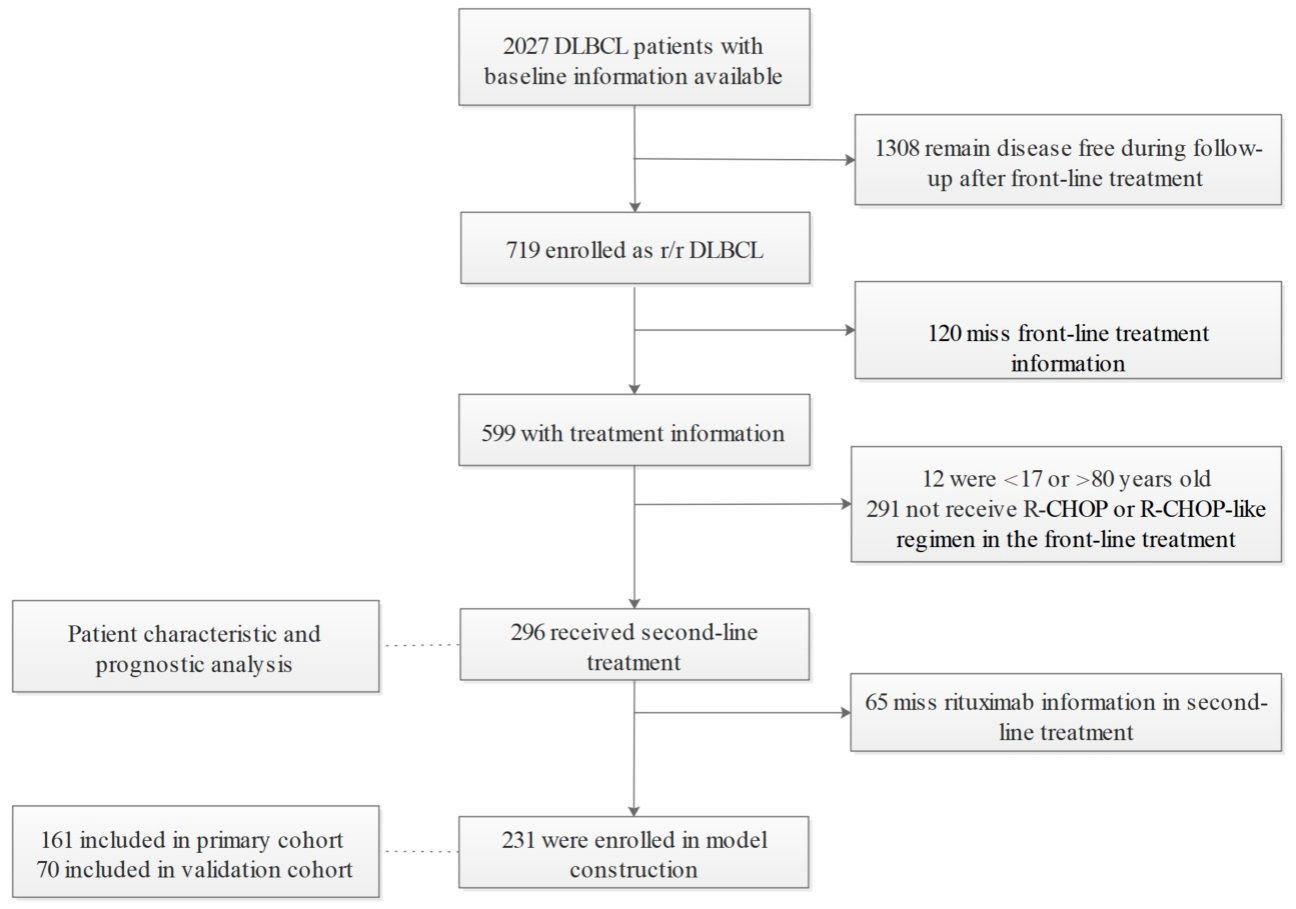
Patient selection.

Among 2027 DLBCL patients diagnosed according to the WHO classification of Tumors of Hematopoietic and Lymphoid Tissue (2008) 20 who were enrolled between January 2006 and December 2017 at the Cancer Hospital, Chinese Academy of Medical Sciences & Peking Union Medical College. 719 had relapsed or refractory disease. Relapse is defined as disease progression after an initial CR or partial response (PR) in the context of first-line treatment. Refractory disease referred to less than a PR during front-line treatment. After excluding patients missing medical records of front-line treatment (n=120), those older than 80 or younger than 18 years old (n=12), and those without the standard R-CHOP regimen in the first-line setting (n=291), 296 patients who fulfilled the selection criteria were included for analysis and 231 entered the nomogram development cohort. In accordance with the 7:3 division principle, random numbers were generated by a computer to assign patients to the primary (n=161) and validation cohorts (n=70) (Figure 1). This study was approved by the Ethics Committee of the Cancer Hospital, Chinese Academy of Medical Sciences.

### Clinical indicators

The clinical features evaluated included the baseline characteristics (gender, age, Ann Arbor stage, ECOG PS score, extranodal involvement, B symptom, bulky disease, IPI index, serum β2-microglobulin [β2-MG] level, neutrophil-to-lymphocyte ratio [NLR], lymphocyte-to-monocyte ratio [LMR], platelet-lymphocyte ratio [PLR], and serum concentrations of LDH), treatment-related information (first-line treatment, second-line treatment, and response to treatment), and r/r features (time and sites of progression/recurrence). Response was assessed per the International Working Group criteria 21. Progression-free survival (PFS) was calculated from front-line treatment administration to a documented disease progression/recurrence. The patients were categorized according to their response to front-line treatment and PFS: R1 (absence of a PR during front-line treatment), R2 (initial CR/unconfirmed CR [CRu]/PR, PFS≤12 months), R3 (initial CR/CRu/PR, PFS >12 but ≤24 months), and R4 groups (initial CR/CRu/PR, PFS>24 months). OS referred to the interval between the initiation of second-line treatment and the time of death or until the last follow-up.

### Nomogram construction and validation

Univariate and multivariate Cox proportional hazards models were performed and identified the independent prognostic indicators, which were then applied to develop a nomogram. Cox regression analysis was performed and the total score of an individual case was assumed as an independent factor. The receiver operating characteristic (ROC) curve and calibration curve were applied for model validation. A concordance index (C index) was estimated from the area under the ROC curve (AUC) and a calibration plot was used to assess the agreement between the predicted and observed survival probabilities in the internal and external validation cohorts.

### Statistical analysis

The chi-squared test was used to compare the efficacy between the different second-line treatment groups. Kaplan-Meier curves and the log-rank test were used for the univariate survival analysis. Cox proportional hazards regression and ordinal regression were used to identify the prognostic indicators. A nomogram based on the Cox model parameters was further developed and a backward stepwise selection process was used to obtain the final model. The data analysis was conducted with IBM SPSS Statistics, Version 21.0, and the H_misc_, rms, survival ROC package in R, version 3.0.2 (http://www.R-project.org) A two-sided p value<0.05 was considered statistically significant.

## Results

### Patient baseline characteristics of r/r DLBCL

Among the 296 r/r DLBCL patients, 162 (54.7%) were male and 134 (45.3%) were female. The median age at diagnosis was 54 (range 18-80) years. There were 108 (36.5%) older than 60; Seventy-seven (26.0%) patients were categorized as germinal center B-cell (GCB) according to the Hans algorithm 22; 205 (69.3%) patients had ≥2 extranodal site involvement; 86 (29.1%) patients had B symptoms; and 77 (26.0%) had bulky masses. There were 125 (42.2%) and 171 (57.8%) patients categorized as limited and advanced disease, respectively. Among those with limited stage disease, 56 patients received radiotherapy. A majority (83.1% [246/296]) of the patients had a good ECOG PS score of 0-1, while 16.9% (50/296) had a score of ≥2.

### Survival analysis, nomogram construction, and internal validation

For patients alive at the last follow-up, the overall median follow-up time was 13.0 months (interquartile range [IQR, defined as the first and third quartiles], 6.0-38.0 months). The 3-year and 5-year OS rates for the entire group were 26.2% and 17.5%, respectively. The patients in the nomogram development cohort (n=231) were randomized into the primary (n=161) and validation (n=70) cohorts.

Factors potentially affecting patient survival in the nomogram development cohort were first identified with the univariate analysis: B symptoms (p<0.001), ECOG PS (p=0.050), response to first-line treatment (p=0.001), r/r patterns (p=0.002), involvement in progression/recurrence (nodal vs extranodal vs both) (p=0.043), invasion on progression/recurrence (new vs original vs both) (p=0.056), and rituximab in second-line treatment (p<0.001). Then the multivariate analysis performed in the primary cohort (n=161) confirmed that the ECOG PS (p=0.001), response to first-line treatment (p=0.001), invasion on progression/recurrence (new vs original vs both) (p=0.023), and rituximab in second-line treatment (p=0.006) were independent risk factors associated with patient outcomes.

**Fig 2:**
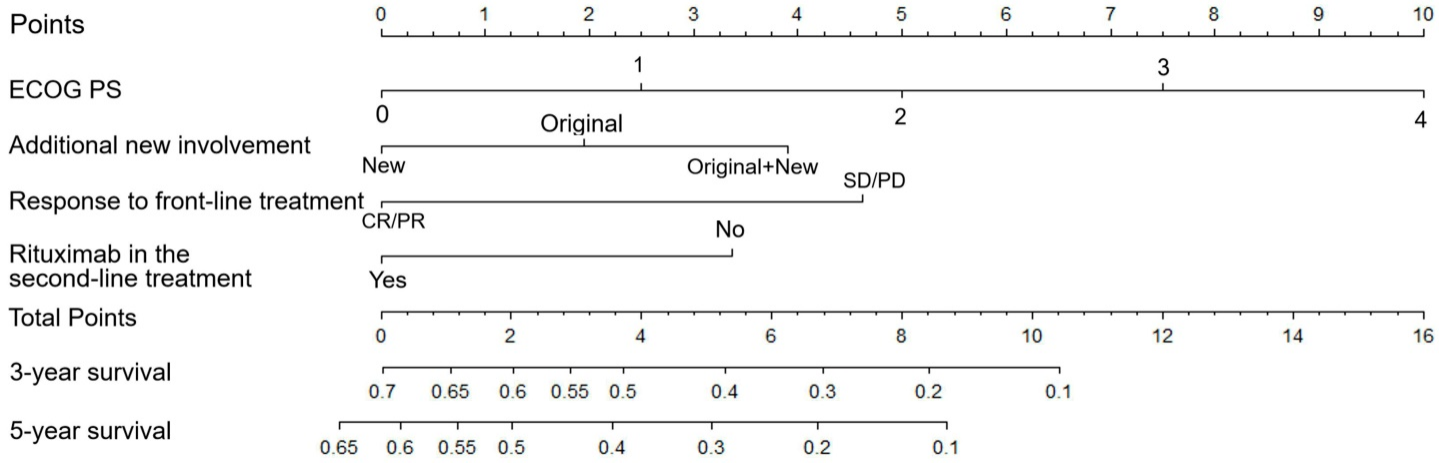
Nomogram of patients with r/r DLBCL. To use the nomogram, the value attributed to a patient is located on each variable axis, and a line is drawn upward to determine the points for each variable value. The sum of the score is located on the total points axis, and a line is then drawn downward to the survival axis to determine the 3- and 5-year OS probability.

Subsequently, the aforementioned four independent prognostic parameters were applied for nomogram construction (Table 1 and Figure 2). The value on the variable axis attributed to an individual case was located and a vertical line was drawn upward from the value on the variable axis to determine the total points assigned to a patient, which enabled estimate of the OS rates on the survival axis. Internal validation showed good agreement between the nomogram-predicted and actual 3-year OS rates from the calibration curve. The C index was 0.70 and the AUC in the internal validation cohort was 0.73.

### External validation and risk group classification

External validation was conducted using a calibration plot with an AUC value of 0.71. An optimal agreement between the actual data and the nomogram-predicted 3-year OS likelihood was also observed. These validation results confirmed the reliability and discriminative ability of the developed nomogram.

**Fig 3:**
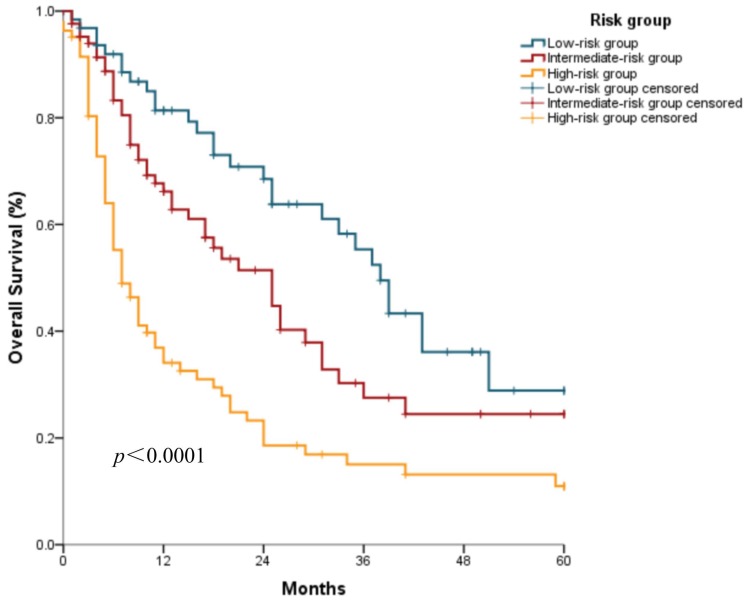
Kaplan-Meier survival curves of the nomogram construction cohort according to the three risk groups defined by the nomogram.

Based on the developed nomogram, three discrete risk groups were determined by the total points: the low-risk group, intermediate-risk group, and high-risk group (Table 2). The median OS was 38.0 (95% confidence interval [CI]: 33.0-43.0) months, 25.0 (95% CI: 16.7-33.3) months, and 7.0 (95% CI: 4.7-9.3) months, respectively, in the low-risk, intermediate-risk, and high-risk groups (Figure 3).

### Relapsed or refractory features, second-line treatment, and risk factors

According to the r/r patterns, 32.8% (97/296), 33.8% (100/296), 18.9% (56/296), and 14.5% (43/296) of the patients were categorized as R1, R2, R3, and R4. Upon disease progression/recurrence, 268 patients had radiological records and 232 had second-line treatment information available at our center. Among them, 39.9% (107/268) had progression/recurrence at the nodal sites, 42.9% (115/268) at the extranodal sites, and 17.2% (46/268) at both sites; 35.1% (104/268) of the patients suffered recurrence at the original sites, 43.6% (129/268) had additional site involvement, and 11.8% (35/268) had progression/recurrence at both the original and additional sites. A total of 43 (16.0%) patients had central nervous system (CNS) involvement after failing the front-line R-CHOP regimen. Among 183 patients with medical records at our center for response to second-line treatment, 96 (52.5%) responded to second-line treatment with 39 (21.3%) CR and 57 (31.1%) PR; 23 (12.6%) received SD and 64 (35.0%) experienced PD. Response to second-line treatment is listed in Table 3. Of those with refractory disease or relapse in the first year from the initiation of treatment (n=133), 57 (42.9%) responded to second-line treatment, with 24 (18.0%) CR/CRu and 33 (24.8%) PR.

In the second-line setting, 45.3% (105/232) of the patients had rituximab-containing treatment and 54.7% (127/232) without rituximab. second-line chemotherapies were grouped by major types: DICE (cisplatin, ifosfamide, etoposide, dexamethasone; n=76), GDP(I) (gemcitabine, dexamethasone, cisplatin, [ifosfamide]; n=46), IC(E) (ifosfamide, carboplatin, [etoposide]; n=38), methotrexate (MTX)-containing regimen (n=20), and GemOx (gemcitabine and oxaliplatin; n=15) were the most frequently used regimens in the second-line setting. MTX was mostly combined with ifosfamide/vincristine/dexamethasone/gemcitabine. Only five patients had lenalidomide in their second-line treatment and none had ibrutinib. No significant difference in efficacy was found among the different regimens. We further divided the regimens into 4 categories based on the number of chemotherapeutic drugs and rituximab use: 1-2 cytotoxic drugs with or without rituximab and ≥3 cytotoxic drugs with or without rituximab. The efficacy of second-line treatment is listed in Table 3. The univariate ordinal regression to filter factors correlated with the response to second-line treatment based on p value were r/r patterns, prior response to front-line treatment, bulky disease at diagnosis and second-line treatment regimens. Decreased ORR of second-line treatment was found for those with initial bulky disease than those without (30.4 vs. 89.3%, p<0.001) as well as nonresponders (28.2 vs. 65.9%, p<0.001) compared with those with a previous response to R-CHOP regimen. We then conducted a multivariate ordinal regression analysis to verify the significant predictors for second-line treatment efficacy, identifying prior response to front-line treatment (odds ratio=4.50, 95% CI: 1.84-11.27, p=0.001) and bulky disease at diagnosis (odds ratio=0.36, 95% CI: 0.182-0.702, p=0.003) as the two independent factors.

## Discussion

A nomogram is a visual format of a statistical predictive model that permits improved predictive accuracy for clinical outcomes compared with the former prognostic scales through calculating the cumulative effect of each independent variable 23. A wide variety of nomograms were previously developed for malignacies and some were indicated for DLBCL 18, 19, 24-30, incorporating clinicopathological parameters to predict the survival of patients (Supplementary Table). Although some of these nomograms were dedicated to incorporating NLR and inflammatory cytokines into nomogram construction for accurate survival prediction 28-30, they were all indicated for de novo patients with front-line treatment. Furthermore, some of the above mentioned DLBCL-specific nomograms have not yet been validated in an external cohort 28. Additionally, a nomogram previously proposed based on r/r DLBCL in our center included patients in the pre- and post-rituximab era, without treatment information in the second-line setting. In this study, we presented four independent indicators of the survival outcome of r/r DLBCL patients after R-CHOP failure and combined them to construct a prognostic nomogram for this population showing favorable validity and reliability. External validation was performed with this newly-developed nomogram. In addition, we demonstrated the efficacy of variable second-line therapies in real-world practice in the rituximab era and addressed parameters of predictive potential. Taken together, this study established a prognostication tool for risk stratification allowing for individualized risk-adapted therapy in patients with r/r DLBCL.

The prognosis of the patients in the present study is relatively poor, with 3-year and 5-year OS rates of 26.2% and 17.5%, respectively, compared with previous data regarding r/r populations 5, 31, 32. This survival disparity could be due to the real-life practice nature of this study and rituximab use in the first-line setting. To date, robust evidence that has addressed the survival benefit of second-line treatment was affected by prior rituximab treatment, with a 3-year EFS of 21% in a rituximab-exposed group and 47% in a rituximab-naive group 5. Moreover, although the majority of the patients had a good PS, 16.9% had ECOG PS scores ≥2, which could not only reflect the comorbidities and tolerance of the patients but also affect the patients' and/or physicians' treatment preferences in the first-line setting.

The outcomes are affected by various factors, as was addressed by the saaIPI, with identical risk factors to the aaIPI 11, 33. Demonstrated as a predictive tool for PFS and OS in r/r DLBCL patients, the saaIPI divided patients into 3 well-defined risk groups: low risk (0 factor), intermediate risk (1 factor), and high risk (2 or 3 factors). Although the saaIPI was developed before the rituximab era 6, it remains predictive in the rituximab era. Nevertheless, it is noteworthy that uniform treatment in the front-line setting was absent in previous studies, and exploration prognostic indicators with front-line R-CHOP regimens are of significance. More specifically, the saaIPI comprises 3 risk factors 33, PS, LDH level, and disease stage, applied at the time of relapsed or primary refractory disease. Different from the saaIPI, in which the LDH level was determined before second-line treatment, we included LDH at diagnosis for analysis to reflect the biological nature of the disease, given the level of LDH could be affected by prior treatment. Likewise, although the PS level before second-line treatment is a routine consideration for treatment decisions, we applied ECOG PS at diagnosis to analyze the impact of PS on front-line treatment and subsequent response.

In general, the rate of relapse was between 30-40% in DLBCL, with an additional 10% of patients not responding to initial treatment. The relationship between early relapse and patient prognosis has been firmly recognized. In the PARMA study, patients experiencing early relapses less than 12 months after diagnosis had the same poor prognosis as those with primary refraction. Moreover, in the published literature 5,31,34-39, patients with early relapses, especially those failing rituximab-containing first-line therapy, had a poor outcome. Generally, primary refractory disease represents the most challenging subtype of DLBCL and is often chemoresistant, leading to significantly shorter OS and PFS compared to those with later relapses 10, 40. In the present study, we demonstrated similar outcomes for this population. More importantly, with the assistance of the developed nomogram, we are capable of better characterizing the most high-risk group.

Although second-line chemotherapy may improve outcomes for patients failing front-line treatment, the ideal second-line regimen remains undetermined 41-42. Two trials were dedicated to the management of r/r patients. Before access to rituximab, the PARMA trial prospectively included 215 patients with relapsed NHL (mostly DLBCL) and established an OS rate of 46% for the ASCT group and 12% for the chemotherapy/radiation alone group, respectively, at a median follow-up of more than 5 years 31. The shortcoming of this trial is that it was conducted in the pre-rituximab era and included young patients. The other trial exerting significant impact is the CORAL study, a phase 3 multicenter prospective study that randomized patients to receive two second-line regimens (R-ICE and R-DHAP). The ORR was 63.5% with R-ICE and 62.8% with R-DHAP and 3-year OS rates were 47% and 51%, respectively 5. In the present study, which was conducted in the context of real-world practice, 52.5% of the r/r patients responded to second-line treatment, with 21.3% CR/CRu and 31.1% PR. The percentages are relatively lower than those in the CORAL study, which could be because all of the patients enrolled in our study were previously exposed to rituximab. Notably, among the patients who responded to second-line treatment, only 7 (7.3%) received ASCT. A recent study compared the efficacy of ofatumumab (O) vs rituximab (R) in combination with cisplatin, cytarabine, and dexamethasone (DHAP) second-line treatment in r/r DLBCL patients with first-line R-CHOP failure. The response rate for O-DHAP was 38% and 42% for R-DHAP, which is comparable to that in our study 43. Based on our research, we conclude that rituximab use in second-line treatment could still benefit patient outcomes after the failure of front-line R-CHOP treatment. In addition, parameters independently affected second-line treatment efficacy were examined, indicating patients with initial bulky disease and less than a PR to first-line treatment would have lower ORR in the second-line setting. These data were consistent with the existing data and added understading for the risk stratification for this population. Based on the newly developed nomogram and the independent indicators to predict the efficacy of second-line treatment, a subset of high-risk patients was determined. For those with factors such as refractory to prior treatment, new additional and original disease involvement, and bulky disease, outcome may be poor and valid treatment options are limited for this population. Thus, novel biological agents are preferred and expected to benefit this population.

As this was a retrospective study, incomplete clinical information may have introduced difficulties and bias to our research. Moreover, the data analyzed in this study were obtained from a patient cohort in a single center with over one-third cases missing information regarding the regimen and the efficacy of second-line treatment. Finally, the prognostic indicators for the efficacy of second-line therapy have yet not been validated in an independent cohort and with the emergence of novel treatment modalities and targeted drugs, the prognostication will soon be renewed. Future studies should be designed to incorporate more novel biomarkers for better risk stratification and risk-adapted treatment.

## Conclusions

In summary, our study addressed the prognostic parameters of survival and response to second-line treatment for r/r DLBCL patients in the post-rituximab era. Additionally, we established the first prognostic nomogram for patients with r/r DLBCL. This work lays the foundation for clinical practice, from survival prediction to second-line treatment guidance. With the development of gene-expression analysis and innovative approaches, an improved understanding of prognosis could boost a risk-adapted treatment paradigm, which could hopefully contribute to improving the devastating outcomes in r/r DLBCL.

## Supplementary Material

Supplementary table.

## Figures and Tables

**Table 1:** Prognostic index model of four risk factors

Variable		Score
Rituximab in the second-line setting	Yes	0
	No	3
ECOG PS	0	0
	1	2
	2	5
	3	8
	4	10
Response to front-line treatment	CR/PR	0
	SD/PD	5
Invasion on progression/recurrence	New involvement	0
	Original involvement	2
	Both	4

Abbreviations: ECOG PS, Eastern Cooperative Oncology Group performance status; CR, complete remission; PR, partial remission; SD, stable disease; PD, progressive disease.

**Table 2:** Predicted outcomes of the respective risk groups according to the developed nomogram.

Risk group	Score	Overall survival (months)	95% CI (months)
Low	0-4	38	33.0-43.0
Intermediate	5-7	25	16.7-33.3
High	>8	7	4.7-9.3

Abbreviations: CI, confidence interval.

**Table 3:** Response to different second-line treatment in the patients with r/r DLBCL

Response	1-2 drug (n=119)			≥3 drug (n=98)	
	Without R n (%)	With R n (%)		Without R n (%)	With R n (%)
CR/CRu	10 (17.5)	9 (19.1)		6 (20.7)	14 (28.0)
PR	13 (22.8)	18 (38.3)		8 (27.6)	18 (36.0)
SD	9 (16.7)	4 (8.5)		5 (17.2)	5 (10.0)
PD	25 (43.9)	16 (34.0)		10 (34.5)	13 (26.0)
NA	7	8		2	17
Total	64	55		31	67

Abbreviations: NA, not accessible; R, rituximab; CR, complete remission; PR, partial remission; SD, stable disease; PD, progressive disease.
