# Supplementary material for: Prognostic Nomogram and Predictive Factors in Refractory or Relapsed Diffuse Large B-Cell Lymphoma Patients Failing Front-Line R-CHOP Regimens
Source: J Cancer. 2020 Jan 14;11(6):1516–24. doi: 10.7150/jca.36997 (PMC6995391; doi:10.7150/jca.36997)
Supplement: Supplementary file 1 — Supplementary table. [file jcav11p1516s1.pdf]

Supplementary Table: Comparison of previous literature and the reports with our study.

PREVIOUS PUBLICATION ON NOMOGRAM	RESPONSE
Prognostic nomogram for diffuse large B-cell lymphoma incorporating the International Prognostic Index with interim-positron emission tomography findings. Chow A, Phillips M, Siew T, Cull G, Augustson B, Ward M, Joske D.<br>Intern Med J. 2013 Aug;43(8):932-9	This nomogram was indicated for de novo patients with immunochemotherapy in the front-line treatment. Although this nomogram incorporated PET-CT into nomogram construction, several biomarkers such as gene rearrangement and double expression were not included. Besides, the sample size was relatively small with 76 patients included and no validation was performed.
Nomogram incorporating clinicopathological parameters to predict the survival of patients with mantle cell lymphoma. Zhu Y, Xu W, Zheng X, Zheng Z. J Investig Med. 2019 Feb;67(2):331-337.	This is a nomogram incorporating clinicopathological parameters to predict the survival of patients with mantle cell lymphoma. Similarly, we used the same method for nomogram construction in a different population: r/r DLBCL patients. Moreover, we also explored the parameters influencing the response to salvage treatment, which added clinical value of clinical guidance of our study.
Nomogram to predict thymoma prognosis: A population-based study of 1312 cases.<br>Zhao M, Yin J, Yang X, Jiang T, Lu T, Huang Y, Li M, Yang X, Lin M, Niu H, Zhan C, Feng M, Wang Q. Thorac Cancer. 2019 May;10(5):1167-1175.	Although this nomogram was constructed based on a large cohort (n=1312). nomogram developing cohort was performed using the SEER database which prevented us from reaching a more precise result because lack of detailed treatment-related information.
Prognostic nomogram for overall survival in previously untreated patients with extranodal NK/T-cell lymphoma, nasal-type: a multicenter study. Yang Y, Zhang YJ, Zhu Y, Cao JZ, Yuan ZY, Xu LM, Wu JX, Wang W, Wu T, Lu B, Zhu SY, Qian LT, Zhang FQ, Hou XR, Liu QF, Li YX. Leukemia. 2015 Jul;29(7):1571-7.	This nomogram was developed for the prediction of overall survival in patients with extranodal NK/T-cell lymphoma, nasal-type. Although this nomogram was based on 1383 patients with NKTCL treated at 10 participating institutions between 2000 and 2011, chemotherapy detailed information was absent, which may somewhat limited the generalisability of this finding.

A new prognostic model using the NCCN-IPI and neutrophil-to-lymphocyte ratio in diffuse large B-cell lymphoma. Go SI, Park S, Kim JH, Kim HR, Kim M, Moon K, Seo J, Lee GW. Tumori. 2018 Aug;104(4):292-299.	This nomogram was indicated for de novo patients with immunochemotherapy in the front-line treatment. Although this nomogram incorporated neutrophil-to-lymphocyte ratio into nomogram construction, biomarkers such as gene rearrangement and double expression were not included. Besides, validation in an independent cohort was warranted.
Prognostic nomogram incorporating inflammatory cytokines for overall survival in patients with aggressive non-Hodgkin's lymphoma.<br>Zhong H, Chen J, Cheng S, Chen S, Shen R, Shi Q, Xu P, Huang H, Zhang M, Wang L, Wu D, Zhao W. EBioMedicine. 2019 Mar;41:167-174.	This nomogram was developed for non-Hodgkin's lymphoma based on a large population with external validation. Athough it seemed to be a useful tool with validity, it can still be optimized by including more DLBCL-specific clinical indicators for nomogram development.
Prognostic Nomogram for Overall Survival in Patients with Diffuse Large B-Cell Lymphoma. Han Y, Yang J, Liu P, He X, Zhang C, Zhou S, Zhou L, Qin Y, Song Y, Sun Y, Shi Y. Oncologist. 2019 Apr 5. pii: theoncologist.2018-0361	This *Ref19* in this manuscript. We revised the manuscript by adding comparison in the discussion. This DLBCL-specific prognostic nomogram was constructed based on the DLBCL database in our centre, collected from June 2006 to December 2012. First, this nomogram was used for newly diagnosed patients. Second, this study included nearly half of patients without rituximab use in the front-line treatment. Thus, it can help to characterize a prognosis of de novo DLBCL instead of r/r DLBCL patients.
A prognostic nomogram constructed for relapsed or refractory diffuse large B-cell lymphoma patients. Jiang S, Qin Y, Liu P, Yang J, Yang S, He X, Zhou S, Gui L, Zhang C, Zhou L, Sun Y, Shi Y. Asia Pac J Clin Oncol. 2019 Jul 1.	The aim of this retrospective study was to find the clinical independent indicators for r/r patients treated with standard first-line anthracycline-based chemotherapy and optimize the risk stratification. However, the fact that this study population spans for a long time period may cause bias. Moreover, treatment information in the salvage setting had not been available yet.
Overall, multiple prognostic models have been developed in NHL and some of them were constructed in de novo DLBCL patients treated with R-CHOP regimen. However, there is no existing prognostic model for r/r DLBCL patients. The visual format of nomogram indicates a statistical predictive model which can determine how many points are attributed for each variable value. The nomograms permit improved predictive accuracy for clinical outcomes when compared with the former prognostic systems.	
